# Magnetic resonance imaging based finite element modelling of the proximal femur: a short-term in vivo precision study

**DOI:** 10.1038/s41598-024-57768-7

**Published:** 2024-03-25

**Authors:** Kadin B. Majcher, Saija A. Kontulainen, David A. Leswick, Allan T. Dolovich, James D. Johnston

**Affiliations:** 1 Department of Mechanical Engineering, University of Saskatchewan, 57 Campus Drive, Saskatoon, SK S7N 5A9 Canada; 2 College of Kinesiology, University of Saskatchewan, 87 Campus Drive, Saskatoon, SK S7N 0W6 Canada; 3 Division of Biomedical Engineering, University of Saskatchewan, 57 Campus Drive, Saskatoon, SK S7N 5A9 Canada; 4 Department of Medical Imaging, University of Saskatchewan, 103 Hospital Drive, Saskatoon, SK S7N 0W8 Canada

**Keywords:** Mechanical engineering, Biomedical engineering

## Abstract

Proximal femoral fractures are a serious life-threatening injury with high morbidity and mortality. Magnetic resonance (MR) imaging has potential to non-invasively assess proximal femoral bone strength in vivo through usage of finite element (FE) modelling (a technique referred to as MR-FE). To precisely assess bone strength, knowledge of measurement error associated with different MR-FE outcomes is needed. The objective of this study was to characterize the short-term in vivo precision errors of MR-FE outcomes (e.g., stress, strain, failure loads) of the proximal femur for fall and stance loading configurations using 13 participants (5 males and 8 females; median age: 27 years, range: 21–68), each scanned 3 times. MR-FE models were generated, and mean von Mises stress and strain as well as principal stress and strain were calculated for 3 regions of interest. Similarly, we calculated the failure loads to cause 5% of contiguous elements to fail according to the von Mises yield, Brittle Coulomb-Mohr, normal principal, and Hoffman stress and strain criteria. Precision (root-mean squared coefficient of variation) of the MR-FE outcomes ranged from 3.3% to 11.8% for stress and strain-based mechanical outcomes, and 5.8% to 9.0% for failure loads. These results provide evidence that MR-FE outcomes are a promising non-invasive technique for monitoring femoral strength in vivo.

## Introduction

Osteoporosis, through its association with age-related fractures, is one of the most common causes of longstanding pain, functional impairment, disability, and death in elderly populations, and a major contributor to medical care costs worldwide^1,2^. Hip fracture, in particular, is a serious life-threatening injury, with fracture of the proximal femoral neck, intertrochanteric and/or shaft regions typically occurring from a sideways fall from standing height. Mortality after hip fracture is high (~ 10%) in the immediate post-fracture period, and remains higher than that of the general population^1,3–5^. For these reasons, effective intervention strategies to reduce the risk of hip fracture at both individual and population levels are warranted.

It is well established that exercise training is beneficial for improving bone strength (i.e., bone’s load carrying capacity or failure load), particularly when starting during early adolescence^6^. With exercise training, bone strength may be maintained (or increased) and hip fracture risk may be reduced in old age. In order to identify specific exercise protocols which reduce hip fracture risk, non-invasive in vivo estimates of proximal femoral strength during adolescence and early adulthood are required.

Dual energy X-ray absorptiometry (DXA) is a two-dimensional (2D) imaging technique offering measures of areal bone mineral density (aBMD) of the proximal femur. The technique is low dose (0.14 µSv^7^), and thus is suitable for adolescents and young adults^8^. DXA-based aBMD measures of the proximal femur offer modest-to-strong agreement with experimentally-derived failure load (fall configuration: R^2^ ranging from 0.41 to 0.92^9–12^; stance configuration: R^2^ ranging from 0.42 to 0.71^13,14^) (for a detailed overview, see summary table in^15^). DXA though offers representations of complex 3D structures as 2D projection images, and thus cannot distinguish between cortical or trabecular bone geometry or material properties, each of which independently contribute to proximal femoral strength^16^. Quantitative computed tomography (QCT) is a three-dimensional (3D) imaging technique offering measures of volumetric BMD (BMD) of both cortical and trabecular bone. On its own, QCT measures of proximal femoral geometry and density offers modest predictions of failure load (fall: R^2^ = 0.19^9^; stance: R^2^ = 0.66^17^). However, when combined with computational finite element (FE) modelling (a method referred to as QCT-FE), the approach offers stronger agreement with experimentally-derived failure load (fall: R^2^ ranging from 0.73 to 0.90^12,18–21^; stance: R^2^ ranging from 0.63 to 0.95^17,19–22^). QCT, however, exposes participants to higher levels of ionizing radiation at the radiosensitive pelvic region (e.g., 2900 µSv from Khoo et al.^23^), which some may argue is ethically unacceptable for growing adolescents and fertile young adults. Accordingly, the QCT-FE technique is typically applied with elderly adult populations. Recently, FE combined with magnetic resonance (MR) imaging (referred to as MR-FE) has seen application for identifying failure regions as well as assessing hip strength of exercise groups engaging in different levels of physical activity (high-impact, odd-impact, repetitive-impact, high-magnitude, non-impact)^24–26^. The key benefits of MR is that it offers multi-planar 3D images and nonionizing radiation of the radiosensitive pelvis (and thus has potential for studying adolescents and young adults). Current research suggests that MR-FE is an accurate tool for estimating mechanical failure loads of the proximal femur with strong agreement with experimentally obtained values (fall: R^2^ = 0.85)^27^. To date, there has only been one study which assessed the in vivo precision error of MR-FE; however, this study focused on whole-bone stiffness and elastic modulus for a small region of interest (ROI)^28^. Currently, the measurement repeatability of MR-FE mechanical outcomes (specifically bone stress and failure load) has not been reported at critical failure regions for fall and stance loading configurations.

Knowledge of the measurement error is important to establish the repeatability of the technique. Specifically, an understanding of the precision error is critical as it identifies parameters which may be best suited for future research related to MR-FE. Relatedly, knowledge of precision error can be used to determine the least significant change (LSC). The International Society of Clinical Densitometry recommends estimating the LSC to determine if observed skeletal differences are true and greater, with 95% confidence, than the measurement error^29^. LSC is estimated using the root-mean squared coefficient of variation (RMS-CV%) multiplied by an adjusting z-score (2.77 × RMS-CV% for 95% confidence) and is an important quantitative metric to ensure changes are sufficiently larger than the precision error^30,31^. LSC is suitably important for clinical studies and comparing bone strength differences. To date, LSCs have not been reported for MR-FE derived mechanical outcomes.

The objective of this study was to characterize the in vivo measurement precision of MR-FE mechanical outcomes of the proximal femur (bone stress and failure load, specifically) for configurations simulating fall and stance loading.

## Methods

### Participants

Thirteen healthy participants (5 males and 8 females) with ages ranging from 21 to 68 years (median age: 27 years), and weights ranging from 54 to 105 kg (median: 70 kg), were recruited as part of a previous study at the University of Saskatchewan^32^. Participant information is presented in Table 1. Study approval was obtained from the University of Saskatchewan Biomedical Research Ethics Board. All study procedures were conducted in accordance with the guidelines approved by the Biomedical Research Ethics Board and the Declaration of Helsinki. Informed consent was obtained from all study participants.

**Table 1:** Participant characteristics.

Participant	Sex	Age (years)	Weight (kg)
1	M	41	63
2	F	23	105
3	F	22	54
4	M	23	64
5	M	27	70
6	M	28	90
7	F	21	70
8	F	26	63
9	F	32	72
10	F	68	59
11	F	31	54
12	M	24	95
13	F	32	80

### MRI scan parameters

MRI scans of the left proximal femur were obtained from a previous research study^32^. Axial images (relative to the orientation of the participant) of the hip were obtained using a clinical 1.5 T scanner (Magnetom Avanto, Siemens, Germany) with a 6-channel body array coil positioned over the hip region. Each participant was positioned supine with their left leg extended and externally rotated 15˚. Scanned image volumes included ~ 2 cm superior to the femoral head and concluded ~ 5 cm inferior to the lesser trochanter. A T1-weighted turbo spin echo sequence was used with the following parameters: TR 616 ms, TE 12 ms, 2 excitations, 180˚ flip angle, 0.45 × 0.45 mm in plan pixel size, 4 mm slice thickness, ~ 4.5 min scan time, ~ 40 images. Each participant was scanned three times with repositioning done following a short walk between repeat scans.

### Image analysis

Intensity shading inhomogeneity, commonly known as “bias field”, was present in the original MRI scans^33^. An open-source software platform for medical imaging (3D Slicer) was used in conjunction with a non-parametric, non-uniform intensity normalization module (N4ITK) to interactively correct the image inhomogeneity^34,35^. Each original scan of the proximal femur was individually loaded and processed using the correction module. Images were then qualitatively checked for shading improvement.

Using commercial software (Analyze 12.0: Mayo Foundation, Rochester, MN, USA), MRI scans were semi-automatically segmented to delineate the proximal femur from surrounding soft tissue. Each image slice was segmented in the transverse plane followed by manual correction. Subject-specific thresholds (defined via the half-maximum height, HMH) method approach were used to define the periosteal boundary and separate it from the soft tissue^36,37^. The thresholds were defined at a site approximately 2 cm below the lesser trochanter on the femoral shaft^32^. All segmentations were performed by a single researcher (K.B.M.). The original discrete MRI scans and segmentations were reformatted via cubic interpolation to create isotropic cubic arrays (from 0.45 × 0.45 × 4 mm to 0.45 × 0.45 × 0.45 mm). Following interpolation, binary masks were adjusted in the coronal plane to reduce delineation precision errors caused by participant repositioning between scans.

**Figure 1:**
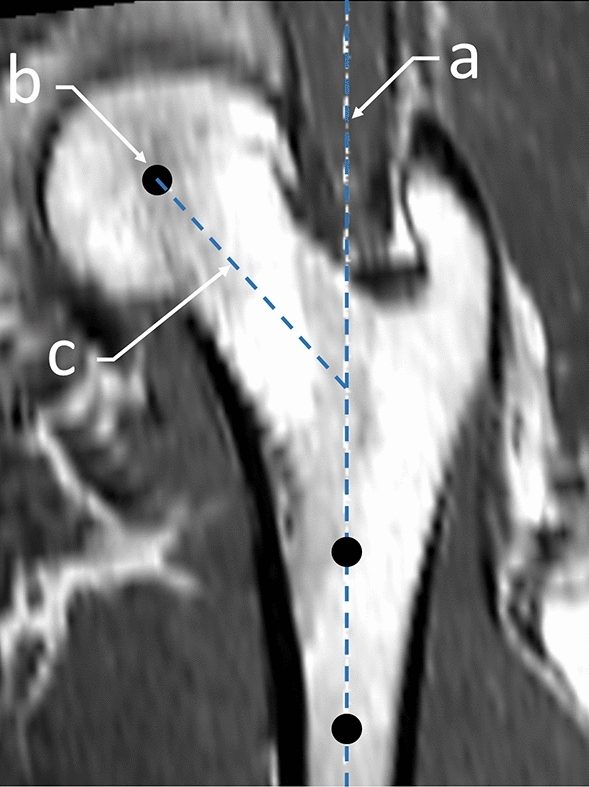
MRI scans were aligned into a common 0° orientation (shown) and then rotated into fall and stance configurations prior to FE model generation. Using the segmented mask data, the long axis of the femur (aka shaft axis) (**a**) was defined by identifying the line-of-best-fit through centroids of axial slices distal to the greater trochanter. The center of the femoral head (**b**) was identified by fitting a sphere to the surface of the head via a variant of the iterative closest point algorithm. A vector corresponding with the neck was also defined by identifying the line-of-best-fit through centroids of slices in an axial-oblique orientation. This vector was then projected to a plane containing the shaft axis and center of the femoral head. The neck axis (**c**) was defined as the projected vector passing through the femoral head and intersecting with the shaft axis. This configuration was used to define the common 0° orientation with the shaft axis aligned vertically and the neck axis aligned with 0° internal/external rotation.

**Figure 2:**
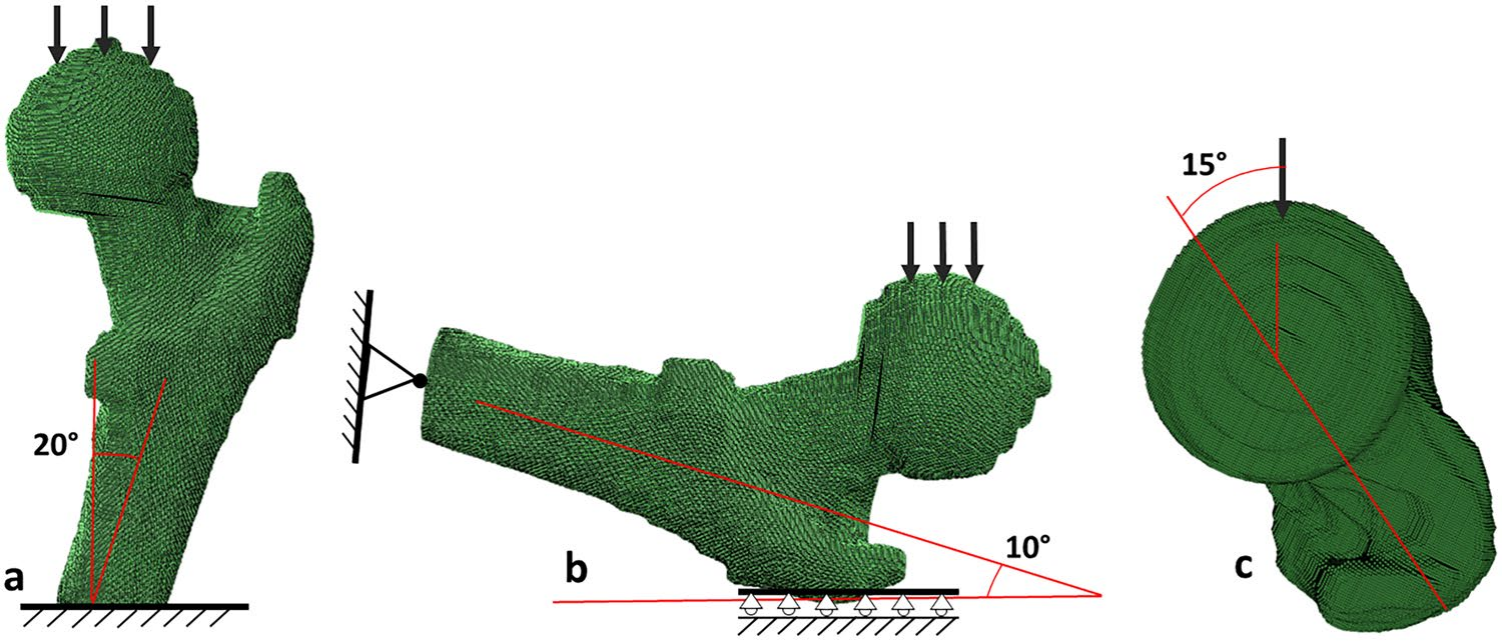
Stance and fall loading configurations of the FE models. The shaft long axis was rotated 20° from the vertical and an initial distributed load applied over the femoral head for the stance models (**a**). For the fall configuration, the femoral shaft was tilted 10° with respect to the ground (**b**) and the neck axis was internally rotated 15° (**c**). The distal shaft was constrained with a hinge-type boundary condition (prohibiting displacements but allowing rotations), and the greater trochanter nodes were restrained in the direction of the distributed load.

Image volumes (scans and masks) were aligned into fall and stance loading orientations using custom coding (Matlab 2018a; MathWorks, Natick, MA, USA), as per previous proximal femoral FE studies^26,38^. Using mask data, this process involved identifying the center of the femoral head by fitting a sphere to the surface of the head via a variant of the iterative closest point algorithm^39^. The long axis of the femur (aka shaft axis) was defined by identifying the line-of-best-fit through centroids of axial slices distal to the greater trochanter. A plane was then fit to the shaft axis and the center of the femoral head. A vector corresponding with the neck was also defined by identifying the line-of-best-fit through centroids of slices in an axial-oblique orientation. This vector was then projected to the plane containing the shaft axis and center of the femoral head. The neck axis was defined as the projected vector passing through the femoral head and intersecting with the shaft axis. This configuration was used to define the common 0° orientation with the shaft axis aligned vertically and the neck axis aligned with 0° internal/external rotation (Fig. 1). From here the images were rotated to the stance configuration (shaft long axis rotated 20° from vertical^38^) and fall configuration (shaft long axis tilted 10° with respect the ground with the neck axis internally rotated 15°^26^) (Fig. 2).

### FE modelling

FE models representative of stance and sideways fall loading configurations were generated from the realigned MRI volumes and segmentations. Using custom algorithms (Matlab), we converted each voxel into an 8-noded hexahedral element with dimensions corresponding to the 0.45 mm voxel size. Bone material properties were assumed to be linearly elastic and isotropic, with the elastic moduli of each voxel computed from the image intensity. Voxel-specific bone volume fraction’s (BVF) were computed from the image intensity via BVF = 1 − (*Int*_*voxel*_/*Int*_*max*_), as per^40^. A custom MRI phantom was used to verify that a linear relationship exists between image intensity and BVF (R^2^ > 0.99) (Supplementary Material). Imaged BVF was converted to elastic moduli (E) via the equation *E* = 12.9[1.08(1-Int_voxel_/Int_max_)]^2^, where *Int*_*voxel*_ is the intensity of each voxel and *Int*_*max*_ is the maximum fat intensity in the scan. This equation was based upon Öhman et al.^41^ density-modulus equation for the proximal femur, combined with conversion equations linking BVF, apparent density and ash density^42,43^. A Poisson’s ratio of 0.3 was assumed for all elements^44^.

Nodal connectivity and material properties of the proximal femur were imported into Abaqus (version 6.13, Providence, RI, USA) for loading and analysis (Fig. 2). For the loading configurations, we applied a distributed load over the femoral head. The distal shaft was fully constrained for the stance models as in previous studies^20,21,38^. For the sideways fall, a hinge-type boundary condition was applied on the distal shaft, and the most lateral nodes of the greater trochanter were fully constrained in the direction of the force^21,26,45^. For both the stance and sideways fall configurations, an arbitrary load of 1 body weight was applied (arbitrary in that the linearity of the models allowed for the results to be scaled).

### FE outcomes

**Figure 3:**
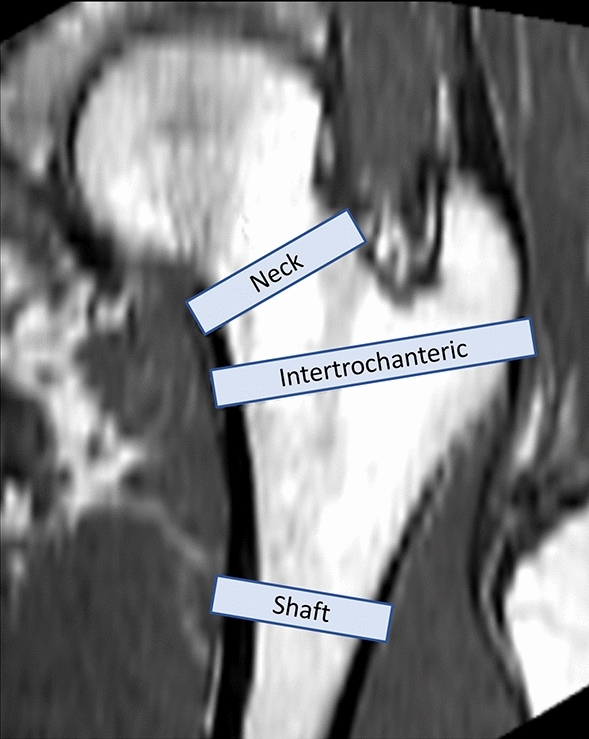
FE outcomes were reported at 4.5 mm thick regions at the femoral neck (center of the femoral neck axis between the head center and vertical shaft axis), intertrochanteric (bi-sector of the angle between the neck and shaft), and shaft (20 mm below the inferior edge of the lesser trochanter).

The FE outcomes were analyzed at 4.5 mm thick anatomical regions of interest (Fig. 3) at the neck, intertrochanteric, and shaft. The regions were selected based on common critical failure regions and automatically defined using anatomical landmarks and custom coding (Matlab)^38,45^. For each region and orientation, the mean von Mises stress, von Mises strain, principal stresses, and principal strains were calculated. The principal stresses and strains were used to derive failure loads from four different failure criteria, including the von Mises yield, brittle Coulomb-Mohr (BCM), normal principal, and Hoffman criteria stress and strain analogs^19,20,46–48^. Failure theories were assessed at the three regions of interest for each configuration. The applied force was linearly scaled to determine the failure load which would cause 5% of contiguous elements to fail.

Strain and equivalent stress limits were used for cortical and trabecular bone. We assigned bone a tensile strain limit of 7000 μstrain^49,50^ and a compressive strain limit of 10,000 μstrain^41^. The equivalent stress limits were assigned by multiplying the strain limits by the respective element’s elastic modulus^46^. The tensile and compressive strain limits (*ε*_*yt*_*, ε*_*yc*_), and stress limits (*σ*_*yt*_, *σ*_*yc*_) were related using the ratios *ε*_*yt*_/*ε*_*yc*_ and *σ*_*yt*_/*σ*_*yc*_, being equal to 0.7^20,51^.

### Statistical analysis

We assessed short-term in vivo precision errors of each outcome using RMS-CV% (short-term refers to the case where measurements are acquired over a time period of less than 1 month, as per Bonnick et al.^31^)^52^. With 13 participants scanned 3 times, this provided 26 degrees-of-freedom (DOF = # participants * (# scans–1)), which met recommendations by Glüer et al.^52^. With this DOF, we established a precision error with an upper 90% confidence limit less than  ~ 30%. We report mean values for each outcome. Short-term precision was also assessed in absolute terms using the root mean square standard deviation (RMS-SD) of the 3 repeat measures.

## Results

### Regional means

For the fall configuration, RMS-CV% precision errors of the regional unadjusted stress and strain measures averaged 7.9% and ranged from 5.3% to 11.7% (Table 2). For the stance configuration, RMS-CV% precision errors of the regional stress and strain measures averaged 7.8% and ranged from 3.3% to 11.8%. RMS-CV% for the strain measures ranged from 7.0% to 11.8%, and 3.3% to 7.9% for the stress measures. Regional stress/strain precision errors appeared similar between the femoral neck, intertrochanteric, and shaft regions.

**Table 2:** Precision results for the MR-FE mechanical outcomes for the fall and stance loading configuration (13 participants, 3 scans each, 26 degrees of freedom).

Orientation	Outcome	Neck					Intertrochanteric					Shaft				
		Mean	Max	Min	RMS-SD	RMS-CV%	Mean	Max	Min	RMS-SD	RMS-CV%	Mean	Max	Min	RMS-SD	RMS-CV%
Fall	von Mises stress [MPa]	4.7	6.9	3.3	0.3	5.4	2.8	4.2	1.8	0.1	5.3	3.4	5.1	1.6	0.2	5.5
	von Mises strain [μstrain]	1021.1	2547.2	465.0	140.7	9.0	2151.6	7540.9	404.3	424.1	11.5	513.2	851.3	165.7	60.3	11.7
	Maximum principal stress [MPa]	1.9	2.9	1.1	0.1	6.2	1.9	2.2	0.8	0.1	6.2	1.4	3.3	0.8	0.1	6.2
	Minimum principal stress [MPa]	− 3.2	− 2.4	− 5.2	0.2	6.1	− 1.7	− 1.0	− 2.6	0.1	6.4	− 1.7	− 0.8	− 3.2	0.1	6.7
	Maximum principal strain [μstrain]	645.6	1466.9	298.5	81.2	9.6	1249.2	4239.4	258.8	159.7	8.8	412.9	649.7	132.4	41.8	9.5
	Minimum principal strain [μstrain]	− 995.7	− 460.9	− 2729.6	152.4	9.4	− 2258.6	− 408.4	− 7903.6	259.1	9.0	− 399.0	− 133.6	− 655.1	35.0	9.1
Stance	von Mises stress [MPa]	1.5	2.5	0.9	0.1	7.2	0.7	1.1	0.5	0.0	4.8	1.8	3.0	1.1	0.1	6.0
	von Mises strain [μstrain]	250.4	354.4	144.3	26.2	9.9	224.7	606.3	89.9	43.3	11.4	253.4	392.3	168.9	36.2	11.8
	Maximum principal stress [MPa]	0.3	0.6	0.1	0.0	7.2	0.3	0.3	0.1	0.0	7.2	0.2	0.8	0.2	0.0	6.9
	Minimum principal stress [MPa]	− 1.2	− 0.8	− 2.0	0.1	7.9	− 0.6	− 0.4	− 1.0	0.0	6.9	− 1.4	− 1.0	− 2.0	0.1	3.3
	Maximum principal strain [μstrain]	158.3	245.7	92.1	14.8	9.0	201.4	620.4	60.3	26.2	8.7	149.2	240.4	88.9	9.4	7.0
	Minimum principal strain [μstrain]	− 255.2	− 138.6	− 380.8	23.6	9.3	− 189.2	− 87.6	− 390.0	14.0	8.7	− 234.5	− 166.1	− 316.4	18.5	7.7

Mean values are presented at three critical regions (neck, intertrochanteric, shaft). Precision is reported using root mean square standard deviations (RMS-SD) and coefficients of variation (RMS-CV%).

### Failure loads

RMS-CV% precision errors for failure loads in the fall configuration averaged 7.5% and ranged from 5.8% to 9.0% (Table 3). RMS-CV% precision errors of failure loads for the stance configuration averaged 7.3% and ranged from 6.4% to 8.1%. Failure load precision errors were  < 8.2% at the femoral neck,  < 9.0% at intertrochanteric region, and  < 8.3% at the shaft (Table 3).

**Table 3:** Precision results for the MR-FE failure loads for the fall and stance loading configuration (13 participants, 3 scans each, 26 degrees of freedom).

Orientation	Outcome	Neck				Intertrochanteric				Shaft			
		Mean [kN]	Range	RMS-SD	RMS-CV%	Mean [kN]	Range	RMS-SD	RMS-CV%	Mean [kN]	Range	RMS-SD	RMS-CV%
Fall	BCM stress	9.4	5.2–14.5	0.7	7.0	12.4	5.0–21.6	1.0	7.3	15.2	8.5–25.5	1.2	7.4
	BCM strain	9.3	5.2–15.0	0.7	7.8	12.4	6.0–24.1	0.8	6.8	14.7	8.3–26.0	1.1	8.2
	Normal principal stress	7.7	3.9–13.8	0.6	8.0	10.2	4.8–18.2	0.8	8.3	16.2	7.0–21.1	1.2	7.1
	Normal principal strain	7.6	3.0–11.0	0.5	5.8	9.8	4.6–18.5	0.6	6.0	16.4	7.1–22.1	1.4	8.0
	von Mises stress	8.5	4.5–13.2	0.6	7.8	11.0	4.6–20.4	0.8	8.1	15.0	8.7–20.8	1.3	8.1
	von Mises strain	8.2	4.1–16.4	0.8	8.2	10.2	6.3–19.5	1.0	9.0	14.5	9.7–20.4	1.2	8.3
	Hoffman stress	9.6	5.4–13.9	0.7	6.9	12.5	5.6–20.3	0.8	6.5	15.5	9.6–23.8	1.0	6.4
	Hoffman strain	8.6	5.5–13.8	0.7	7.3	11.2	6.0–19.3	0.9	7.6	13.8	8.3–18.4	1.1	8.0
Stance	BCM stress	14.7	10.3–19.7	1.1	7.1	12.8	6.5–20.4	0.9	7.3	14.7	10.6–24.1	1.1	7.6
	BCM strain	14.3	9.5–19.7	1.1	7.6	11.9	5.4–18.3	0.9	7.8	14.8	8.3–25.9	1.1	7.4
	Normal principal stress	10.9	6.9–17.5	0.9	7.7	13.5	5.7–20.7	0.7	6.8	12.0	6.7–18.0	0.9	7.1
	Normal principal strain	11.0	6.2–14.7	0.9	7.9	13.8	5.5–19.6	1.0	6.9	12.3	8.4–20.7	0.9	7.3
	von Mises stress	11.4	7.0–15.8	0.9	8.0	11.4	6.2–15.0	0.8	6.9	13.6	9.2–20.7	0.9	6.4
	von Mises strain	11.2	6.7–15.9	0.9	8.1	11.6	6.2–15.5	0.8	7.4	13.8	9.3–21.2	1.0	7.1
	Hoffman stress	15.1	8.2–18.8	1.1	6.7	14.7	7.0–20.1	1.0	6.7	16.1	9.7–22.8	1.2	7.3
	Hoffman strain	14.4	8.2–18.8	0.9	6.6	14.7	7.7–21.0	1.1	7.9	14.5	8.7–19.8	1.2	8.0

The mean failure loads [kN] to cause 5% of the elements to fail at three critical regions (neck, intertrochanteric, shaft) are presented. Precision of each failure criterion is reported using root mean square standard deviations (RMS-SD) and coefficients of variation (RMS-CV%).

## Discussion

This study characterized short-term in vivo precision errors of MR-FE outcomes of the proximal femur for two loading configurations and three regions. To our knowledge, this is the first study to report FE precision errors at the neck, intertrochanteric, and shaft regions using MR-FE. This study complements existing studies which focused on evaluating differences in MR-FE outcomes between groups and provides indication of measurement error.

Generally, the von Mises stress, principal stresses, principal strains, and failure loads had similar precision errors (RMS-CV% < 8.3%), except for the von Mises strain criterion which was higher (RMS-CV% < 11.8%). The high measurement error of the von Mises strain outcomes may be attributed to the small strain values, whereby a small variation resulted in a large precision error. Our FE-based in vivo precision error results are similar (though slightly higher) to previous QCT-FE findings at the knee, which had an average RMS-CV% of  < 6%^53^. Additionally, MR-FE precision errors for the two configurations are comparable with no substantial differences. In comparison to an MR precision study of bone morphology (e.g., cortical thickness)^32^, which used the same scan data evaluated here, reported precision errors were smaller (< 7.1%) than the errors reported here. Though, our study considered FE outcomes of 3D volumetric ROI’s whereas Johnston et al.^32^ reported metrics based on single 2D image slices.

To sufficiently recommend a best-suited failure criterion for future MR-FE studies, various parameters including precision error (RMS-CV%), explained variance (R^2^), and ability to capture changes or differences are needed for consideration. With regards to the presented precision errors, the four failure theories assessed in this study were similar and provided measurement errors  ≤ 9.0%. Though, a large range of estimated failure loads may indicate a more sensitive criterion for identifying differences in bone strength for MR-FE. In this case, BCM (stress and strain) generally had the largest failure load ranges. In line with this finding, and comparable measurement error with other failure criteria, BCM may best characterize hip strength. Future research is needed to evaluate experimentally-derived failure loads against MR-FE derived estimates acquired via various failure theories to identify the best-suited criterion.

**Figure 4:**
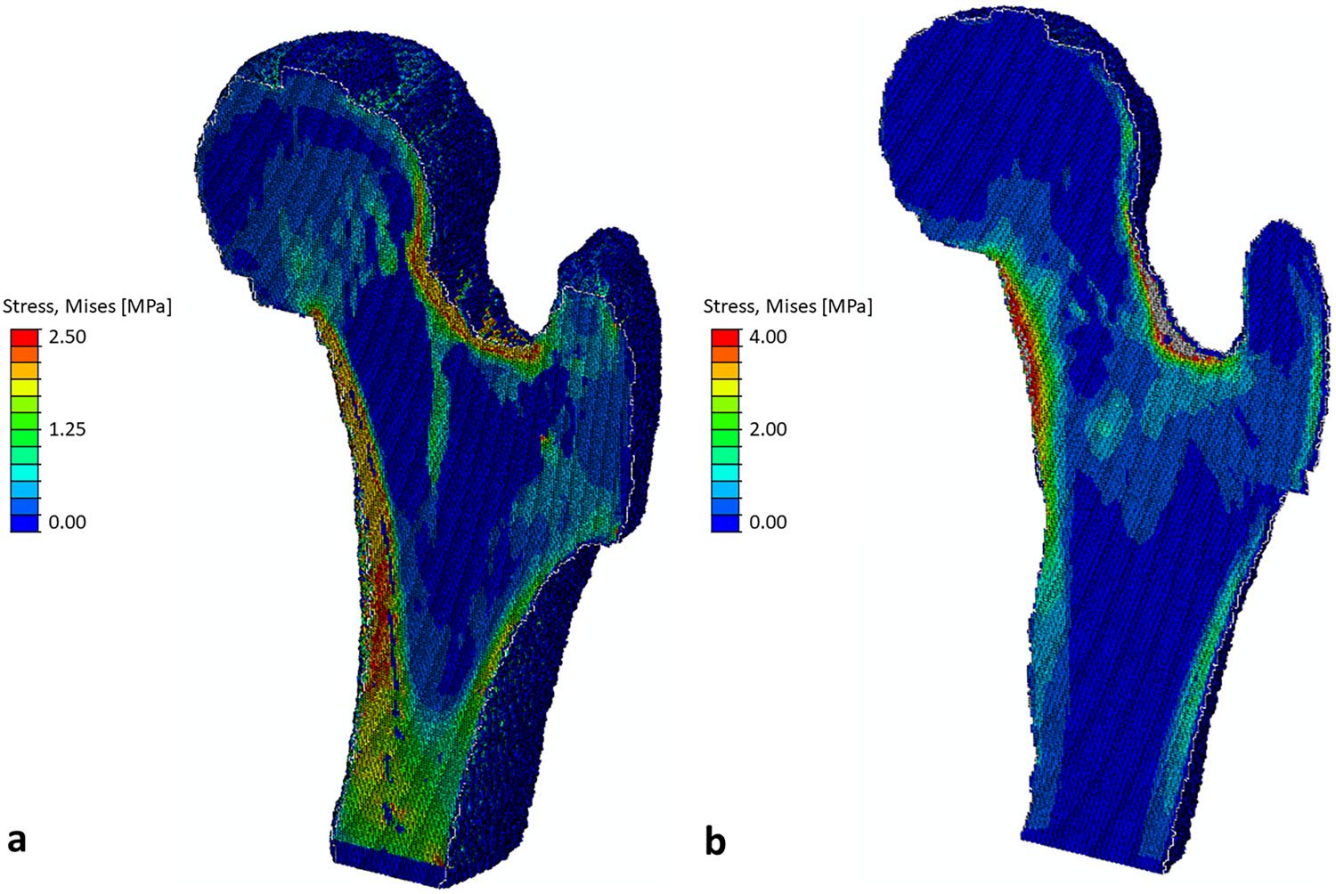
Example of the internal von Mises stress distribution under an applied load of 1 body weight for the stance (**a**) and sideways fall (**b**) loading configurations.

Numerical failure load results from this study are similar to those published in previous research^25^. The estimated failure loads from our study, focused on a young adult population, ranged from 3.0 to 16.4 kN at the neck in the fall configuration. Previous experimental studies found failure loads ranging from 5.2 kN to 8.5 kN for the same site and configurations^45,54,55^; though, these findings were specific to elderly adult (> 70 years of age) cadaveric femurs. As adult femurs are approximately twice as strong as elderly adult femurs^54^, our results may be comparable. Our failure load findings though are specific to the applied criteria (e.g., 5% of elements failing). A lower percentage of failed elements would lead to lower failure loads approaching experimental findings. Accordingly, further validation research is needed identifying specific modelling approaches (e.g., failure criterion, percentage of failed elements) best-suited for predicting failure of the proximal femur. Of note, stress and strain outcomes presented in this paper are presented for measurement repeatability only. The applied force magnitude of 1 body weight was arbitrary and lower than estimated failure loads. The lower applied load can explain lower stress values (Table 2, Fig. 4) in comparison to other MR-FE research (e.g., Abe et al.^26^ used an impact force ~ 8 × body weight).

This research has strengths requiring consideration. First, with MR-FE, each voxel of the proximal femur was modeled as a hexahedral element, allowing us to preserve the cortical detail from the scans. Conversely, using tetrahedral elements requires intensive surface smoothing and careful strategies to map elastic moduli to elements. The surface smoothing process inherently incorporates voxels inside and/or outside the original image mask, which may lead to loss of femoral detail. Secondly, we applied a custom algorithm to automatically align MR scans into the fall and stance loading configurations, which reduced variation between repeat scans, leading to a lower precision error. Third, we report precision errors at three clinically relevant regions^56,57^ for the two commonly studied loading configurations in the literature. The inclusion of different regions and loading configurations provides information of regional precision. Fourth, we have used a conservative sample size (13 participants, 39 scans, 26 DOF) to establish precision errors with an upper 90% confidence interval limit of  ~ 30%, as proposed by Glüer et al.^52^. Although our study did not exactly meet the DOF recommendations (28 DOF), the upper 90% confidence limit with our DOF (31%) is comparable to recommendations (30%).

With regards to limitations, first, due to the large slice thickness (4 mm), the true 3D geometry of the femur was difficult to capture and resulted in a jagged structure. The large slice thickness may have resulted in under/over estimation of bone strength as critical bone features may not have been captured in the original scans. To more accurately characterize the shape of the proximal femur, our original scans consisting of 37 slices were interpolated to 329 slices. This approach led to a more correct shape, but small variations in material properties were not truly captured. Second, due to the poor signal-to-noise ratios on some scans, it was difficult to identify the periosteal surface within the intertrochanteric region. To segment, we defined the boundary using semi-automatic region growing and subject-specific thresholds (HMH)^37^, followed by manual segmentation where needed. Operator judgment had an influence on femoral segmentations and may have induced error. Third, presented MR-FE models of the proximal femur were not validated against mechanical testing, unlike previous QCT-FE studies^12,17–22^. To address this, we adopted similar boundary and loading conditions as previous studies and compared our numerical results^25,26,38^. However, it would be beneficial to validate MR-FE derived estimates of bone failure load, along with corresponding failure criteria, reported here. Fourth, our study assessed the short-term precision errors of relatively young adults (median age: 27 years), making it difficult to generalize our results beyond the studied age group. Still, our study provides insight into MR-FE measurement precision and supports the application of MR-FE for monitoring bone strength differences. Fifth, in this study we applied short-term precision errors to estimate LSC. Glüer et al.^30^ though advises to use long-term precision errors (i.e., measures taken over at least 1 year) in the LSC calculation to account for factors such as scanner calibration, drift and differences in operator technique. Unfortunately (and in line with Bonnick et al.^31^), we found that the logistical difficulties in performing a long-term precision study, compounded with the need to apply linear regression to account for biological changes due to growth and development, made the approach unfeasible. Accordingly, it is important to be cognizant that the LSC presented here may be underestimated.

In conclusion, this study found that short-term precision errors were less than 11.8% for the two loading configurations. Precision errors ranged from 3.3% to 11.8% for regional stress and strain mean outcomes, and 5.8% to 9.0% for failure loads. This is the first study to assess the short-term in vivo precision error of MR-FE outcomes for fall and stance loading configurations at the proximal femur. Results from this study demonstrate that MR-FE outcomes are a promising non-invasive technique for monitoring femoral strength in vivo and may guide future studies in their assessment of femoral strength.

### Supplementary Information

Supplementary Information.

## Data Availability

The datasets generated during and/or analyzed during the current study are available from the corresponding author on reasonable request.
