# Supplementary material for: Magnetic resonance imaging based finite element modelling of the proximal femur: a short-term in vivo precision study
Source: Sci Rep. 2024 Mar 25;14:7029. doi: 10.1038/s41598-024-57768-7 (PMC10963727; doi:10.1038/s41598-024-57768-7)
Supplement: Supplementary file 1 — Supplementary Information. [file 41598_2024_57768_MOESM1_ESM.pdf]

Supplementary Material

Magnetic Resonance Imaging Based Finite Element Modelling of the Proximal Femur: a Short-Term *in vivo* Precision Study

Kadin B Majcher[1], Saija A Kontulainen[2,3], David A Leswick[4], Allan T Dolovich[1,3], James D Johnston[1,3]

1. *Department of Mechanical Engineering, University of Saskatchewan, 57 Campus Drive, Saskatoon, Saskatchewan S7N 5A9*
2. *College of Kinesiology, University of Saskatchewan, 87 Campus Drive, Saskatoon Saskatchewan S7N 0W6*
3. *Division of Biomedical Engineering, University of Saskatchewan, 57 Campus Drive, Saskatoon, Saskatchewan S7N 5A9*
4. *Department of Medical Imaging, University of Saskatchewan, 103 Hospital Drive, Saskatoon Saskatchewan S7N 0W8*

We verified that a linear relationship exists between the calculated and known BVF's for our scan parameters and homogeneity corrected images. To test, we created a phantom using 6 jars containing 3 different solid materials simulating bone of known BVF's and oil simulating marrow (Supplementary Figure S1). Solid material was placed in a vial, fully submerged in canola oil, and then placed in a vacuum chamber for degassing. The phantom was scanned with parameters matching the participant hip scans. The phantom scan was corrected for inhomogeneity [1,2], and then interpolated to create an isotropic array (Analyze 12.0). Each slice of the MR scan was then semi-automatically segmented. Manual corrections were applied to remove lodged air bubbles using commercial software (Analyze 12.0).

Using pure canola oil as the maximum scan intensity, we computed the BVF of each material. Plotting the known BVF's against the calculated BVF's (Supplementary Figure S2), it was found that a strong linear relationship exists (coefficient of determination, $R^2 > 0.99$). Results confirmed that the linear relationship used to compute BVF from the MRI intensities remained true following the inhomogeneity correction.

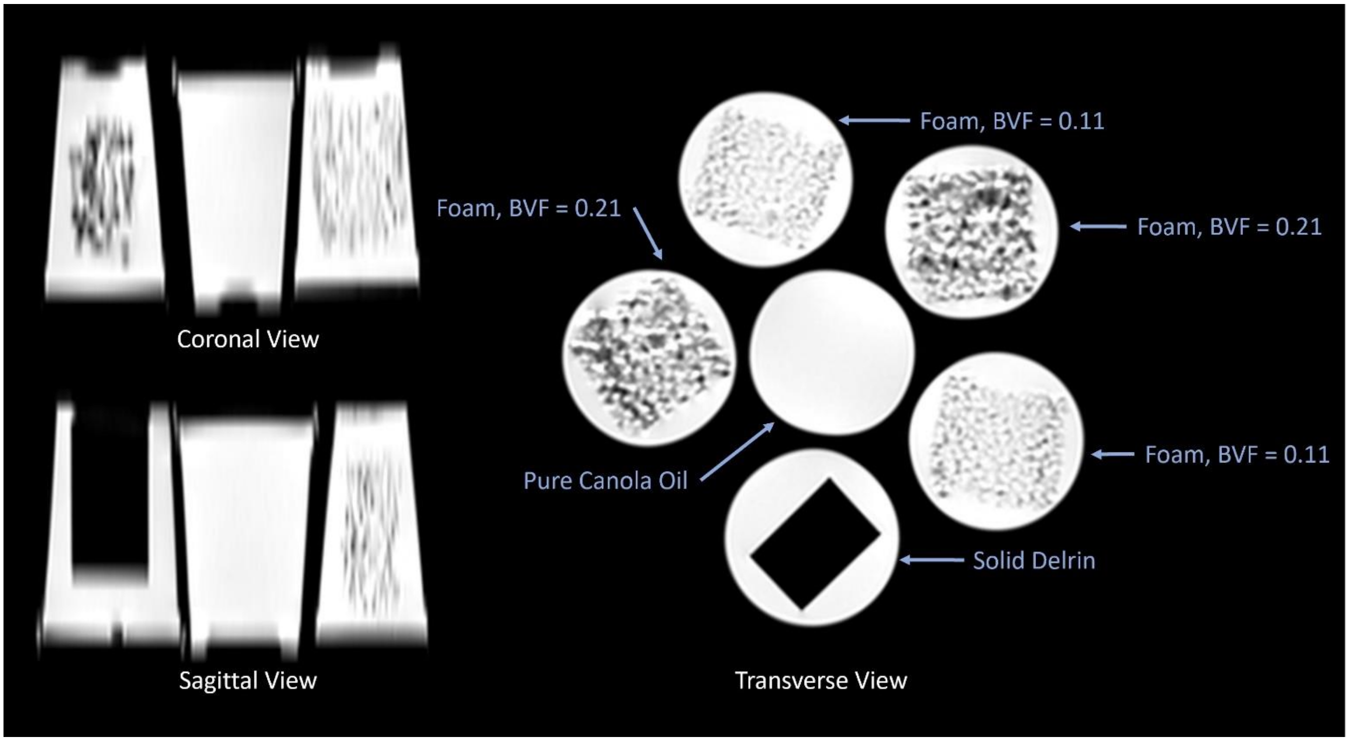

Supplementary Figure S1. Coronal, sagittal and transverse views of the MR phantom. The phantom consisted of pure canola oil, Delrin, 20 PCF foam (BVF = 21%), and 7.5 PCF foam (BVF = 11%).

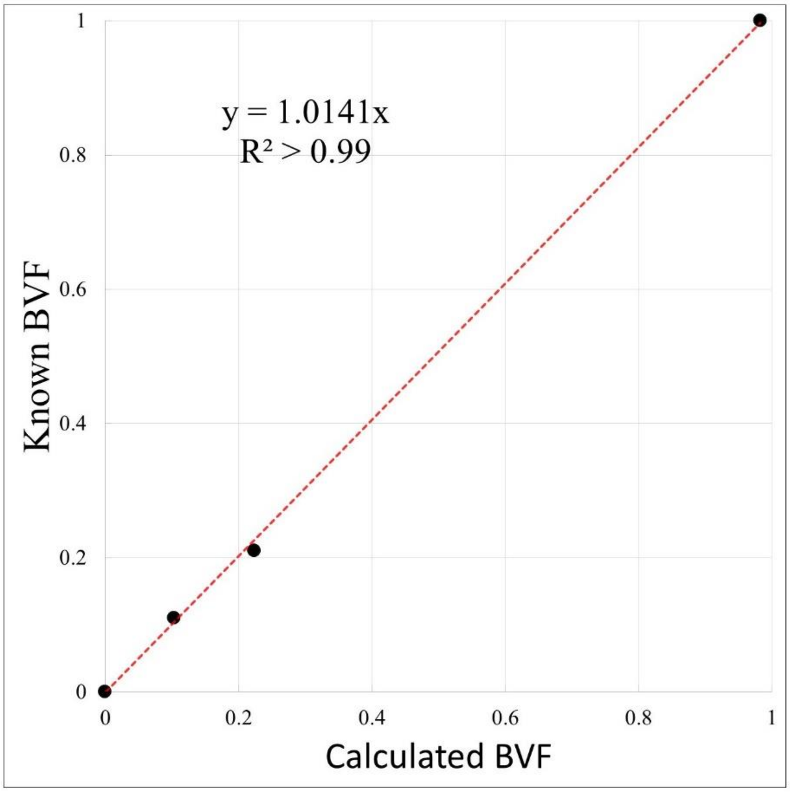

Supplementary Figure S2. Linear regression results for the known and calculated BVF's of the MRI phantom with a best fit line.

Supplementary Material References

1 Fedorov, A. *et al.* 3D Slicer as an image computing platform for the Quantitative Imaging Network. *Magnetic Resonance Imaging* **30**, 1323-1341, doi:https://doi.org/10.1016/j.mri.2012.05.001 (2012).

2 Tustison, N. J. *et al.* N4ITK: Improved N3 Bias Correction. *IEEE Transactions on Medical Imaging* **29**, 1310-1320, doi:10.1109/TMI.2010.2046908 (2010).
